# Supplementary material for: Functional specialization of angular gyrus and precuneus subregions for perspective-guided autobiographical memory retrieval
Source: Imaging Neurosci (Camb). 2026 Jul 27;4:IMAG.a.1313. doi: 10.1162/IMAG.a.1313 (PMC13409283; doi:10.1162/IMAG.a.1313)
Supplement: Supplementary Material [file IMAG.a.1313_supp.pdf]

Supplemental Material

Functional Specialization of Angular Gyrus and Precuneus Subregions for Perspective-Guided Autobiographical Memory Retrieval

Selen Küçüktaş and Peggy L. St. Jacques

Department of Psychology, University of Alberta, Edmonton, AB, Canada

Corresponding Author:
Dr. Peggy L. St. Jacques
Department of Psychology
University of Alberta
P-549 Biological Sciences Building
Edmonton, AB Canada T6G 2R3
Email: peggy.stjacques@ualberta.ca

Supplemental Results: Brain-Behavior Relationship with Categorical ROI Comparison

We conducted exploratory linear mixed-effects models in which ROI was treated as a categorical factor (PGa vs PGp; 7A vs 7M vs 7P) testing Region × fMRI signal × Condition interactions (see Supplemental Tables 17 – 28).

Vividness. During the cue phase, there was a significant condition and percent signal change (fMRI activity) interaction in the AG (see Supplemental Table 17), specifically when comparing the Own Eyes and Observer conditions (*β* = -.143, *SE* = .053, 95% CI [-.25, -.04], *t* (4152.7) = -2.68, *p* = .007). To follow up on the significant interaction, we estimated simple slopes of percent signal change on vividness within each retrieval orientation condition. A significant negative slope was observed in the Observer (*β* = -.134, SE = .037, 95% CI [-.21, -.06], t (4148) = -3.63, p < .001) and Retrieve conditions (*β* = -.078, SE = .037, 95% CI [-.15, -.01], t (4151) = -2.11, p = .035), indicating that greater AG recruitment was associated with reduced vividness. The corresponding slope in the Own Eyes condition (*β* = .008, SE = .039, 95% CI [-.07, .08], t (4144) = .21, p = .834) was not statistically significant. In contrast, there were no interactions between percent signal change when comparing the Own Eyes versus Retrieve (*β* = -.086, SE = .053, 95% CI [-.19, .02], t (4142.8) = -1.63, p = .103) or the Observer versus Retrieve conditions (*β* = .056, SE = .052, 95% CI [-.05, .15], t (4140.6) = 1.08, p = .280).

During the probe phase, there was a significant condition and percent signal change (fMRI activity) interaction in the AG (see Supplemental Table 18), specifically when comparing the Own Eyes and Observer conditions (*β* = .146, *SE* = .055, 95% CI [.04, .26], *t* (4032.5) = 2.64, *p* = .008). To follow up on the significant interaction, we estimated simple slopes of percent signal change on vividness within each retrieval orientation condition. A significant positive slope was

observed in the Observer condition (β = .165, SE = .047, 95% CI [.07, .26], t (87.8) = 3.51, p < .001), indicating that greater AG recruitment was associated with higher vividness. The corresponding slope in the Own Eyes (β = .018, SE = .047, 95% CI [-.08, .11], t (89.6) = .40, p = .693) and Retrieve conditions (β = .091, SE = .047, 95% CI [-.00, .19], t (99.6) = 1.93, p = .057) were not statistically significant. In contrast, there were no interactions between percent signal change when comparing the Own Eyes versus Retrieve (β = .072, SE = .056, 95% CI [-.04, .18], t (4112.9) = 1.29, p = .198) or the Observer versus Retrieve conditions (β = -.073, SE = .056, 95% CI [-.18, .04], t (4119.6) = -1.33, p = .189).

Turning to the precuneus, during the cue phase (see Supplemental Table 19) there was a significant condition and percent signal change (fMRI activity) interaction, specifically when comparing the Own Eyes and Retrieve conditions (β = -.096, *SE* = .035, 95% CI [-.17, .03], *t* (6214.9) = -2.70, *p* = .007). To follow up on the significant interaction, we estimated simple slopes of percent signal change on vividness within each retrieval orientation condition. However, simple effects revealed no significant effect in any of the retrieval orientation conditions (Own Eyes: β = .057, SE = .035, 95% CI [-.01, .13], t (59.9) = 1.61, p = .113; Observer: β = -.002, SE = .035, 95% CI [-.07, .07], t (58.5) = -.06, p = .954; Retrieve: β = -.039, SE = .035, 95% CI [-.11, .03], t (60.2) = -1.10, p = .278). There were no interactions between percent signal change when comparing the Own Eyes versus Observer (β = .072, SE = .056, 95% CI [-.04, .18], t (4112.9) = 1.29, p = .198) or the Observer versus Retrieve conditions (β = -.037, SE = .035, 95% CI [-.11, .03], t (6212.9) = -1.05, p = .292).

During the probe phase, retrieval orientation condition was a significant predictor of vividness ratings in precuneus (see Supplemental Table 20), when comparing the Own Eyes and

Retrieve (β = .065, SE = .024, 95% CI [.02, .11], t(6212.7) = 2.74, p = .006), and Observer and Retrieve conditions (β = .101, SE = .024, 95% CI [.05, .15], t(6218.9) = 4.20, p < .001). Additionally, percent signal change during the cue phase was a significant predictor of vividness in Session 2 (β = .056, SE = .016, 95% CI [.02, .09], t(6276.3) = 3.43, p < .001). However, precuneus subregion, or two-way interactions between variables did not significantly predict vividness ratings in Session 2 (all *p*s > .140).

Emotional Intensity. During the cue phase, there were no significant differences in the AG (all *p*s > .187; see Supplemental Table 21). During the probe phase (see Supplemental Table 22), percent signal change in AG significantly predicted emotional intensity ratings (β = .103, SE = .048, 95% CI [.01, .20], t (35.8) = 2.13, p = .198), indicating increased neural recruitment in AG during the probe phase is associated with higher emotional intensity. There were no other significant predictors or interactions.

Turning to the precuneus, during the cue phase there was a significant condition and percent signal change (fMRI activity) interaction (see Supplemental Table 23), specifically when comparing the Own Eyes and Retrieve (β = .090, *SE* = .036, 95% CI [.02, .16], *t* (5937.3) = 2.51, *p* = .012) and Observer and Retrieve conditions (β = .091, *SE* = .036, 95% CI [.02, .16], *t* (5984.4) = 2.54, *p* = .011). To follow up on the significant interaction, we estimated simple slopes of percent signal change on emotional intensity within each retrieval orientation condition. A significant negative slope was observed in the Retrieve condition (β = -.073, SE = .030, 95% CI [-.13, -.01], t (123) = -2.47, p = .015), indicating that greater precuneus recruitment was associated with lower vividness. The corresponding slope in the Own Eyes (β = .017, SE = .029, 95% CI [-.04, .08], t (119) = .59, p = .558) and Observer conditions (β = .018, SE = .029, 95% CI [-

.04, .08], t (117) = .62, p = .535) were not statistically significant. In contrast, there were no interactions between percent signal change when comparing the Own Eyes versus Observer conditions (β = .001, SE = .036, 95% CI [-.07, .07], t (5806.3) = .03, p = .977).

During the probe phase, within the precuneus there was a significant condition and percent signal change (fMRI activity) interaction (see Supplemental Table 24), specifically when comparing the Observer and Retrieve conditions (β = -.107, *SE* = .035, 95% CI [-.18, -.04], *t* (6135.1) = -3.02, *p* = .003). To follow up on the significant interaction, we estimated simple slopes of percent signal change on emotional intensity within each retrieval orientation condition. However, simple effects revealed no significant effect in any of the retrieval orientation conditions (Own Eyes: β = -.012, SE = .037, 95% CI [-.09, .06], t (55.1) = -.34, p = .733; Observer: β = -.070, SE = .037, 95% CI [-.15, .01], t (57.7) = -1.87, p = .067; Retrieve: β = .037, SE = .037, 95% CI [-.04, .11], t (56.8) = .98, p = .329). There were no interactions between percent signal change when comparing the Own Eyes versus Retrieve (β = -.049, SE = .035, 95% CI [-.12, .02], t (6120.9) = -1.41, p = .158) or the Own Eyes versus Observer conditions (β = -.057, SE = .035, 95% CI [-.13, .01], t (6136.1) = -1.64, p = .101).

Perspective Maintenance. During the cue phase, there was a significant condition and percent signal change (fMRI activity) interaction in the AG (see Supplemental Table 25), specifically when comparing the Own Eyes and Observer (β = -.141, *SE* = .067, 95% CI [-.27, -.01], *t* (4117.9) = -2.09, *p* = .037), and Own Eyes and Retrieve conditions (β = -.162, *SE* = .067, 95% CI [-.29, -.03], *t* (4110.7) = -2.41, *p* = .016). To follow up on the significant interaction, we estimated simple slopes of percent signal change on perspective maintenance within each retrieval orientation condition. A significant positive slope was observed in the Own Eyes

condition (β = .120, SE = .049, 95% CI [.02, .22], t (4106) = 2.43, p = .015), indicating that greater AG recruitment was associated with higher perspective maintenance. The corresponding slope in the Observer (β = -.020, SE = .047, 95% CI [-.11, .07], t (4115) = -.44, p = .662) and Retrieve conditions (β = -.041, SE = .047, 95% CI [-.13, .05], t (4120) = -.88, p = .378) were not statistically significant. In contrast, there were no interactions between percent signal change when comparing the Observer versus Retrieve conditions (β = -.020, SE = .065, 95% CI [-.15, .11], t (4110.4) = -.32, p = .752).

During the probe phase in the AG (see Supplemental Table 26), retrieval orientation was a significant predictor of perspective maintenance ratings, when comparing the Own Eyes and Observer (β = -.329, SE = .053, 95% CI [-.43, -.22], t(4067.8) = -6.16, p = .038), Own Eyes and Retrieve (β = .440, SE = .053, 95% CI [.01, .22], t(4070.2) = 2.07, p < .001), and Observer and Retrieve conditions (β = .111, SE = .053, 95% CI [.34, .55], t(4068) = 8.26, p < .001). AG subregion, percent signal change during the probe phase, or two-way interactions between variables did not significantly predict perspective maintenance ratings in Session 2 (all *p*s > .163).

Turning to the precuneus, during the cue phase there was a significant condition and percent signal change (fMRI activity) interaction (see Supplemental Table 27), specifically when comparing the Own Eyes and Observer conditions (β = -.120, *SE* = .045, 95% CI [-.21, -.03], *t* (6097.4) = -2.67, *p* = .008). To follow up on the significant interaction, we estimated simple slopes of percent signal change on perspective maintenance within each retrieval orientation condition. A significant positive slope was observed in the Own Eyes condition (β = .106, SE = .039, 95% CI [.03, .19], t (76.9) = 2.66, p = .009), indicating that greater precuneus recruitment

was associated with higher perspective maintenance. The corresponding slope in the Observer (β = -.014, SE = .039, 95% CI [-.09, .07], t (76.9) = -.35, p = .727) and Retrieve conditions (β = .039, SE = .039, 95% CI [-.04, .12], t (77) = 1, p = .321) were not statistically significant. In contrast, there were no interactions between percent signal change when comparing the Own Eyes versus Retrieve (β = -.066, SE = .045, 95% CI [-.15, .02], t (6147.3) = -1.48, p = .140) and Observer versus Retrieve conditions (β = .053, SE = .044, 95% CI [-.03, .14], t (6166.3) = 1.20, p = .229).

During the probe phase within the precuneus (see Supplemental Table 28), there was a significant three-way interaction between precuneus subregion, condition, and percent signal change (fMRI activity), specifically when comparing the 7A and 7M activity in the Own Eyes versus Observer (β = .280, *SE* = .119, 95% CI [.05, .51], *t* (6135.8) = 2.35, *p* = .019), and in the Own Eyes versus Retrieve conditions (β = .266, *SE* = .119, 95% CI [.03, .50], *t* (6133.3) = 2.23, *p* = .026). To follow up on the significant interaction, we estimated simple slopes of percent signal change on perspective maintenance in each retrieval orientation condition for each precuneus subregion. Only significant slope was observed in the Own Eyes condition for 7A (β = .238, SE = .078, 95% CI [.08, .39], t (769) = 3.05, p = .002) and 7P activity (β = .118, SE = .059, 95% CI [.00, .23], t (257) = 2, p = .047), indicating that greater 7A and 7P recruitment was associated with higher perspective maintenance. The corresponding slopes in other conditions for each region were not significant (all *p*s > .124).

Supplemental Tables

Supplemental Table 1. Model Predicting Vividness from Left AG (Cue Phase)

Fixed Effect	Estimate (β)	SE	95% CI Lower	95% CI Upper	t-value	p-value
Intercept	3.531	0.072	3.389	3.672	48.92	<.001
Retrieval Condition: Own Eyes	0.0316	0.042	-0.051	0.114	0.748	.455
Retrieval Condition: Retrieve	0.094	0.042	0.011	0.177	2.22	.026
Percent Signal Change (PGa)	-0.015	0.040	-0.092	0.062	-0.39	.696
Percent Signal Change (PGp)	-0.065	0.046	-0.156	0.025	-1.41	.159
PGa x Own Eyes	0.182	0.071	0.042	0.322	2.561	0.010
PGa x Retrieve	0.072	0.069	-0.064	0.209	1.039	0.299
Vividness (S1 covariate)	0.246	0.029	0.189	0.302	8.47	<.001
Emotion (S1 covariate)	0.134	0.018	0.099	0.170	7.51	<.001
Perspective Bias (S1 covariate)	0.040	0.014	0.011	0.069	2.72	.013

Note. The reference level for the retrieval orientation factor is 'Observer'. PGa = percent signal change in left anterior angular gyrus during the cue phase. PGp = percent signal change in left posterior angular gyrus during the cue phase. Session 1 ratings were included as covariates to account for baseline variation in subjective memory experience.

Supplemental Table 2. Model Predicting Vividness from Precuneus (Cue Phase)

Fixed Effect	Estimate (β)	SE	95% CI Lower	95% CI Upper	t-value	p-value
Intercept	3.529	0.072	3.387	3.671	48.76	<.001
Retrieval Condition: Own Eyes	-0.068	0.042	-0.151	0.015	-1.60	.109
Retrieval Condition: Observer	-0.099	0.042	-0.182	-0.016	-2.33	.020
Percent Signal Change (7A)	-0.041	0.058	-0.157	0.073	-0.71	.477
Percent Signal Change (7M)	-0.023	0.034	-0.090	0.044	-0.67	.502
Percent Signal Change (7P)	0.035	0.049	-0.062	0.132	0.70	.482
7A x Own Eyes	0.102	0.083	-0.059	0.265	1.24	.216
7A x Observer	0.028	0.084	-0.136	0.193	0.34	.738
Vividness (S1 covariate)	0.244	0.029	0.187	0.301	8.41	<.001
Emotion (S1 covariate)	0.135	0.018	0.100	0.171	7.55	<.001
Perspective Bias (S1 covariate)	0.040	0.015	0.011	0.069	2.75	.012

Note. The reference level for the retrieval orientation factor is 'Retrieve'. 7A = percent signal change in 7A during the cue phase. 7M = percent signal change in 7M during the cue phase. 7P = percent signal change in 7P during the cue phase. Session 1 ratings were included as covariates to account for baseline variation in subjective memory experience.

Supplemental Table 3. Model Predicting Vividness from Left AG (Probe Phase)

Fixed Effect	Estimate (β)	SE	95% CI Lower	95% CI Upper	t-value	p-value
Intercept	3.526	0.073	3.382	3.670	48.14	<.001
Retrieval Condition: Own Eyes	-0.074	0.042	-0.157	0.009	-1.75	.081
Retrieval Condition: Observer	-0.117	0.042	-0.201	-0.034	-2.76	.006
Percent Signal Change (PGa)	-0.014	0.043	-0.100	0.071	-0.33	.744
Percent Signal Change (PGp)	0.109	0.049	0.012	0.206	2.22	.027
PGp x Own Eyes	-0.057	0.083	-0.220	0.105	-0.69	.490
PGp x Observer	0.079	0.081	-0.080	0.238	0.98	.330
Vividness (S1 covariate)	0.244	0.028	0.188	0.301	8.49	<.001
Emotion (S1 covariate)	0.135	0.018	0.100	0.170	7.51	<.001
Perspective Bias (S1 covariate)	0.041	0.014	0.012	0.070	2.79	.011

Note. The reference level for the retrieval orientation factor is ‘Retrieve’. PGa = percent signal change in left anterior angular gyrus during the probe phase. PGp = percent signal change in left posterior angular gyrus during the probe phase. Session 1 ratings were included as covariates to account for baseline variation in subjective memory experience.

Supplemental Table 4. Model Predicting Vividness from Precuneus (Probe Phase)

Fixed Effect	Estimate (β)	SE	95% CI Lower	95% CI Upper	t-value	p-value
Intercept	3.529	0.072	3.389	3.669	49.39	<.001
Retrieval Condition: Own Eyes	-0.075	0.042	-0.158	0.007	-1.77	.076
Retrieval Condition: Observer	-0.115	0.043	-0.199	-0.032	-2.72	.007
Percent Signal Change (7A)	-0.029	0.063	-0.152	0.094	-0.46	.643
Percent Signal Change (7M)	-0.038	0.032	-0.101	0.025	-1.17	.243
Percent Signal Change (7P)	0.132	0.052	0.029	0.234	2.53	.011
7P x Own Eyes	-0.032	0.060	-0.150	0.085	-0.54	.591
7P x Observer	0.020	0.060	-0.098	0.140	0.34	.731
Vividness (S1 covariate)	0.240	0.028	0.184	0.297	8.37	<.001
Emotion (S1 covariate)	0.137	0.018	0.100	0.172	7.63	<.001
Perspective Bias (S1 covariate)	0.041	0.014	0.012	0.069	2.84	.010

Note. The reference level for the retrieval orientation factor is ‘Retrieve’. 7A = percent signal change in 7A during the cue phase. 7M = percent signal change in 7M during the cue phase. 7P = percent signal change in 7P during the cue phase. Session 1 ratings were included as covariates to account for baseline variation in subjective memory experience.

Supplemental Table 5. Model Predicting Emotional Intensity from Left AG (Cue Phase)

Fixed Effect	Estimate (β)	SE	95% CI Lower	95% CI Upper	t-value	p-value
Intercept	3.326	0.091	3.148	3.505	36.46	< .001
Retrieval Condition: Own Eyes	-0.046	0.043	-0.131	0.038	-1.07	0.284
Retrieval Condition: Observer	-0.090	0.043	-0.175	-0.005	-2.09	0.036
Percent Signal Change (PGa)	-0.062	0.040	-0.142	0.017	-1.54	0.124
Percent Signal Change (PGp)	0.057	0.047	-0.035	0.150	1.22	0.224
PGa x Own Eyes	0.088	0.073	-0.054	0.233	1.22	0.222
PGa x Observer	0.018	0.071	-0.121	0.158	0.26	0.796
Vividness (S1 covariate)	0.054	0.029	-0.002	0.111	1.87	0.072
Emotion (S1 covariate)	0.438	0.033	0.372	0.504	12.95	< .001
Perspective Bias (S1 covariate)	-0.001	0.012	-0.024	0.022	-0.10	0.926

Note. The reference level for the retrieval orientation factor is ‘Retrieve’. PGa = percent signal change in left anterior angular gyrus during the cue phase. PGp = percent signal change in left posterior angular gyrus during the cue phase. Session 1 ratings were included as covariates to account for baseline variation in subjective memory experience.

Supplemental Table 6. Model Predicting Emotional Intensity from Precuneus (Cue Phase)

Fixed Effect	Estimate (β)	SE	95% CI Lower	95% CI Upper	t-value	p-value
Intercept	3.325	0.090	3.147	3.502	36.65	< .001
Retrieval Condition: Own Eyes	-0.038	0.043	-0.123	0.045	-0.90	0.369
Retrieval Condition: Observer	-0.082	0.043	-0.166	0.002	-1.90	0.058
Percent Signal Change (7A)	-0.039	0.060	-0.157	0.078	-0.66	0.509
Percent Signal Change (7M)	-0.042	0.034	-0.111	0.025	-1.22	0.221
Percent Signal Change (7P)	0.048	0.051	-0.051	0.148	0.95	0.342
7A x Own Eyes	0.100	0.102	-0.099	0.300	0.99	0.323
7A x Observer	0.127	0.107	-0.083	0.337	1.18	0.236
7M x Own Eyes	0.045	0.067	-0.086	0.178	0.68	0.499
7M x Observer	0.031	0.069	-0.104	0.166	0.45	0.655
Vividness (S1 covariate)	0.052	0.028	-0.003	0.109	1.83	0.078
Emotion (S1 covariate)	0.439	0.033	0.373	0.506	12.96	< .001
Perspective Bias (S1 covariate)	0	0.012	-0.024	0.020	-0.07	0.946

Note. The reference level for the retrieval orientation factor is ‘Retrieve’. 7A = percent signal change in 7A during the cue phase. 7M = percent signal change in 7M during the cue phase. 7P = percent signal change in 7P during the cue phase. Session 1 ratings were included as covariates to account for baseline variation in subjective memory experience.

Supplemental Table 7. Model Predicting Emotional Intensity from Left AG (Probe Phase)

Fixed Effect	Estimate (β)	SE	95% CI Lower	95% CI Upper	t-value	p-value
Intercept	3.324	0.091	3.145	3.502	36.47	< .001
Retrieval Condition: Own Eyes	-0.043	0.043	-0.128	0.040	-1.01	0.311
Retrieval Condition: Observer	-0.094	0.043	-0.179	-0.009	-2.17	0.030
Percent Signal Change (PGa)	0.077	0.044	-0.009	0.165	1.74	0.081
Percent Signal Change (PGp)	0.027	0.050	-0.072	0.126	0.54	0.591
PGp x Own Eyes	-0.068	0.084	-0.233	0.097	-0.81	0.420
PGp x Observer	-0.007	0.082	-0.169	0.154	-0.09	0.930
Vividness (S1 covariate)	0.054	0.029	-0.002	0.111	1.86	0.072
Emotion (S1 covariate)	0.436	0.033	0.370	0.503	12.93	< .001
Perspective Bias (S1 covariate)	0.00	0.012	-0.023	0.024	0.03	0.977

Note. The reference level for the retrieval orientation factor is ‘Retrieve’. PGa = percent signal change in left anterior angular gyrus during the probe phase. PGp = percent signal change in left posterior angular gyrus during the probe phase. Session 1 ratings were included as covariates to account for baseline variation in subjective memory experience.

Supplemental Table 8. Model Predicting Emotional Intensity from Precuneus (Probe Phase)

Fixed Effect	Estimate (β)	SE	95% CI Lower	95% CI Upper	t-value	p-value
Intercept	3.326	0.090	3.148	3.503	36.73	< .001
Retrieval Condition: Own Eyes	-0.043	0.043	-0.127	0.040	-1.01	0.311
Retrieval Condition: Retrieve	-0.086	0.043	-0.171	-0.001	-1.99	0.046
Percent Signal Change (7A)	-0.038	0.063	-0.163	0.086	-0.607	0.544
Percent Signal Change (7M)	-0.084	0.033	-0.149	-0.018	-2.527	0.012
Percent Signal Change (7P)	0.069	0.053	-0.035	0.174	1.298	0.194
7M x Own Eyes	-0.03	0.05	-0.13	0.07	-0.54	0.587
7M x Retrieve	-0.12	0.05	-0.22	-0.01	-2.23	0.026
Vividness (S1 covariate)	0.05	0.03	0	0.11	1.87	0.072
Emotion (S1 covariate)	0.44	0.03	0.37	0.51	13.04	< .001
Perspective Bias (S1 covariate)	0	0.01	-0.02	0.02	-0.04	0.971

Note. The reference level for the retrieval orientation factor is ‘Observer’. 7A = percent signal change in 7A during the cue phase. 7M = percent signal change in 7M during the cue phase. 7P = percent signal change in 7P during the cue phase. Session 1 ratings were included as covariates to account for baseline variation in subjective memory experience.

Supplemental Table 9. Model Predicting Perspective Maintenance from Left AG (Cue Phase)

Fixed Effect	Estimate (β)	SE	95% CI Lower	95% CI Upper	t-value	p-value
Intercept	3.664	0.086	3.495	3.383	42.53	<.001
Retrieval Condition: Observer	-0.334	0.053	-0.439	-0.229	-6.25	<.001
Retrieval Condition: Retrieve	0.113	0.053	0.008	0.218	2.11	.035
Percent Signal Change (PGa)	0.098	0.050	0	0.197	1.97	.049
Percent Signal Change (PGp)	-0.088	0.058	-0.203	0.026	-1.51	.131
PGa x Observer	-0.209	0.090	-0.386	-0.031	-2.31	0.021
PGa x Retrieve	-0.238	0.090	-0.415	-0.060	-2.63	.009
Vividness (S1 covariate)	0.074	0.025	0.024	0.125	2.90	.004
Emotion (S1 covariate)	0.061	0.022	0.017	0.105	2.71	.007
Perspective Bias (S1 covariate)	0.038	0.018	0.003	0.074	2.14	.043

Note. The reference level for the retrieval orientation factor is 'Own Eyes'. PGa = percent signal change in left anterior angular gyrus during the probe phase. PGp = percent signal change in left posterior angular gyrus during the probe phase. Session 1 ratings were included as covariates to account for baseline variation in subjective memory experience.

Supplemental Table 10. Model Predicting Perspective Maintenance from Precuneus (Cue Phase)

Fixed Effect	Estimate (β)	SE	95% CI Lower	95% CI Upper	t-value	p-value
Intercept	3.660	0.087	3.489	3.831	41.90	<.001
Retrieval Condition: Observer	-0.327	0.053	-0.432	-0.222	-6.10	<.001
Retrieval Condition: Retrieve	0.125	0.053	0.021	0.230	2.35	.019
Percent Signal Change (7A)	0.167	0.073	0.023	0.312	2.27	.023
Percent Signal Change (7M)	-0.020	0.043	-0.105	0.063	-0.49	.627
Percent Signal Change (7P)	-0.058	0.062	-0.180	0.064	-0.93	.352
7A x Observer	-0.224	0.104	-0.429	-0.019	-2.14	.032
7A x Retrieve	-0.214	0.103	-0.417	-0.010	-2.06	.039
Vividness (S1 covariate)	0.073	0.025	0.022	0.123	2.83	.005
Emotion (S1 covariate)	0.063	0.022	0.019	0.107	2.81	.005
Perspective Bias (S1 covariate)	0.039	0.018	0.003	0.075	2.14	.042

Note. The reference level for the retrieval orientation factor is 'Own Eyes'. 7A = percent signal change in 7A during the cue phase. 7M = percent signal change in 7M during the cue phase. 7P = percent signal change in 7P during the cue phase. Session 1 ratings were included as covariates to account for baseline variation in subjective memory experience.

Supplemental Table 11. Model Predicting Perspective Maintenance from Left AG (Probe Phase)

Fixed Effect	Estimate (β)	SE	95% CI Lower	95% CI Upper	t-value	p-value
Intercept	3.657	0.084	3.491	3.823	43.14	<.001
Retrieval Condition: Own Eyes	0.340	0.053	0.234	0.443	6.31	<.001
Retrieval Condition: Retrieve	0.470	0.054	0.364	0.576	8.71	<.001
Percent Signal Change (PGa)	-0.095	0.055	-0.203	0.013	-1.72	.086
Percent Signal Change (PGp)	0.145	0.062	0.023	0.267	2.33	.020
PGp x Own Eyes	-0.099	0.102	-0.299	0.101	-0.07	.332
PGp x Retrieve	-0.105	0.102	-0.307	0.095	-1.03	.302
Vividness (S1 covariate)	0.071	0.025	0.020	0.122	2.77	.006
Emotion (S1 covariate)	0.064	0.022	0.020	0.109	2.86	.004
Perspective Bias (S1 covariate)	0.040	0.018	0.004	0.075	2.22	.035

Note. The reference level for the retrieval orientation factor is ‘Observer’. PGa = percent signal change in left anterior angular gyrus during the probe phase. PGp = percent signal change in left posterior angular gyrus during the probe phase. Session 1 ratings were included as covariates to account for baseline variation in subjective memory experience.

Supplemental Table 12. Model Predicting Perspective Maintenance from Precuneus (Probe Phase: Interaction Model)

Fixed Effect	Estimate (β)	SE	95% CI Lower	95% CI Upper	t-value	p-value
Intercept	3.658	0.081	3.498	3.818	44.81	<.001
Retrieval Condition: Observer	-0.331	0.053	-0.436	-0.226	-6.19	<.001
Retrieval Condition: Retrieve	0.130	0.053	0.026	0.235	2.45	.014
Percent Signal Change (7A)	0.061	0.079	-0.094	0.216	0.77	.442
Percent Signal Change (7M)	-0.183	0.040	-0.263	-0.103	-4.48	<.001
Percent Signal Change (7P)	0.151	0.065	0.022	0.280	2.30	.022
7A x Observer	-0.410	0.167	-0.738	-0.081	-2.44	.014
7A x Retrieve	-0.333	0.161	-0.649	-0.017	-2.07	.015
7P x Observer	0.144	0.123	-0.098	0.386	1.17	0.244
7P x Retrieve	0.036	0.116	-0.191	0.264	0.32	0.753
Vividness (S1 covariate)	0.063	0.025	0.013	0.114	2.47	.014
Emotion (S1 covariate)	0.068	0.022	0.024	0.112	3.05	.002
Perspective Bias (S1 covariate)	0.040	0.018	0.005	0.076	2.24	.035

Note. The reference level for the retrieval orientation factor is ‘Own Eyes’. 7A = percent signal change in 7A during the cue phase. 7M = percent signal change in 7M during the cue phase. 7P = percent signal change in 7P during the cue phase. Session 1 ratings were included as covariates to account for baseline variation in subjective memory experience.

Supplemental Table 13. Model Predicting Vividness from AG (Cue Phase: Categorical Approach for AG Subregions)

Fixed Effect	Estimate (β)	SE	95% CI Lower	95% CI Upper	t-value	p-value
Intercept	3.539	0.074	3.394	3.685	47.66	<.001
Retrieval Condition: Observer	-0.352	0.041	-0.116	0.046	-0.84	.399
Retrieval Condition: Retrieve	0.063	0.041	-0.018	0.144	1.51	.131
AG Subregion[1]	0.003	0.024	-0.043	0.050	0.15	.878
% Sig. Change[2]	-0.057	0.028	-0.114	-0.001	-2.01	.044
AG Subregion x Condition[3]	0.013	0.059	-0.102	0.128	0.22	.823
AG Subregion x Condition[4]	0.000	0.059	-0.114	0.116	0.01	.989
Condition x % Sig. Change[5]	-0.143	0.053	-0.247	-0.038	-2.68	.007
Condition x % Sig. Change[6]	-0.086	0.053	-0.191	0.017	-1.63	.103
AG Subregion x % Sig. Change	-0.021	0.043	-0.106	0.063	-0.49	.621
Vividness (S1 covariate)	0.235	0.030	0.176	0.294	7.84	<.001
Emotion (S1 covariate)	0.132	0.021	0.091	0.174	6.23	<.001
Perspective Bias (S1 covariate)	0.038	0.015	0.008	0.068	2.55	.018

Note. The reference level for the retrieval orientation factor is 'Own Eyes'. Session 1 ratings were included as covariates to account for baseline variation in subjective memory experience.

[1] PGa vs. PGp

[2] Percent signal change in left AG during the cue phase

[3] Interaction between left AG subregions and Condition (Own Eyes vs. Observer)

[4] Interaction between left AG subregions and Condition (Own Eyes vs. Retrieve)

[5] Interaction between Condition (Own Eyes vs. Observer) and percent signal change

[6] Interaction between Condition (Own Eyes vs. Retrieve) and percent signal change

Supplemental Table 14. Model Predicting Vividness from AG (Probe Phase: Categorical Approach for AG Subregions)

Fixed Effect	Estimate (β)	SE	95% CI Lower	95% CI Upper	t-value	p-value
Intercept	3.550	0.075	3.402	3.698	47.10	<.001
Retrieval Condition: Observer	-0.019	0.042	-0.101	0.063	-0.45	.650
Retrieval Condition: Retrieve	0.073	0.042	-0.009	0.156	1.74	.082
AG Subregion[1]	-0.022	0.025	-0.071	0.026	-0.89	.373
% Sig. Change[2]	0.070	0.040	-0.009	0.149	1.72	.091
AG Subregion x Condition[3]	-0.046	0.060	-0.165	0.072	-0.77	.440
AG Subregion x Condition[4]	-0.015	0.060	-0.132	0.103	-0.25	.804
Condition x % Sig. Change[5]	0.146	0.055	0.037	0.255	2.64	.008
Condition x % Sig. Change[6]	0.072	0.056	-0.038	0.184	1.29	.198
AG Subregion x % Sig. Change	0.044	0.047	-0.049	0.137	0.92	.356
Vividness (S1 covariate)	0.233	0.029	0.175	0.291	7.87	<.001
Emotion (S1 covariate)	0.129	0.021	0.088	0.170	6.16	<.001
Perspective Bias (S1 covariate)	0.039	0.015	0.009	0.069	2.54	.018

Note. The reference level for the retrieval orientation factor is ‘Own Eyes’. Session 1 ratings were included as covariates to account for baseline variation in subjective memory experience.

[1] PGa vs. PGp

[2] Percent signal change in left AG during the probe phase

[3] Interaction between left AG subregions and Condition (Own Eyes vs. Observer)

[4] Interaction between left AG subregions and Condition (Own Eyes vs. Retrieve)

[5] Interaction between Condition (Own Eyes vs. Observer) and percent signal change

[6] Interaction between Condition (Own Eyes vs. Retrieve) and percent signal change

Supplemental Table 15. Model Predicting Vividness from Precuneus (Cue Phase: Categorical Approach for Precuneus Subregions)

Fixed Effect	Estimate (β)	SE	95% CI Lower	95% CI Upper	t-value	p-value
Intercept	3.530	0.078	3.376	3.684	44.89	<.001
Retrieval Condition: Observer	-0.034	0.041	-0.115	0.046	-0.83	.404
Retrieval Condition: Retrieve	0.059	0.041	-0.021	0.140	1.45	.148
Precuneus Subregion: 7M	-0.017	0.042	-0.100	0.065	-0.41	.680
Precuneus Subregion: 7P	0.005	0.041	-0.075	0.086	0.14	.887
% Sig. Change[1]	0.048	0.046	-0.042	0.139	1.05	.296
Precuneus Subregion x Condition[2]	0.008	0.059	-0.107	0.124	0.14	.889
Precuneus Subregion x Condition[3]	0.017	0.059	-0.098	0.133	0.30	.765
Precuneus Subregion x Condition[4]	-0.004	0.058	-0.118	0.109	-0.08	.939
Precuneus Subregion x Condition[5]	-0.006	0.058	-0.121	0.107	-0.12	.906
Condition x % Sig. Change[6]	-0.059	0.035	-0.129	0.009	-1.68	.093
Condition x % Sig. Change[7]	-0.096	0.035	-0.167	-0.026	-2.70	.007
Precuneus Subregion x % Sig. Change[8]	0.014	0.040	-0.064	0.093	0.36	.719
Precuneus Subregion x % Sig. Change[9]	0.012	0.041	-0.068	0.092	0.30	.762
Vividness (S1 covariate)	0.234	0.029	0.176	0.292	7.93	<.001
Emotion (S1 covariate)	0.131	0.021	0.088	0.174	6.02	<.001
Perspective Bias (S1 covariate)	0.028	0.015	0.008	0.068	2.53	.018

Note. The reference level for the retrieval orientation factor is ‘Own Eyes’. The reference level for the precuneus subregion factor is ‘7A’. Session 1 ratings were included as covariates to account for baseline variation in subjective memory experience.

[1] Percent signal change in the precuneus during the cue phase
[2] Interaction between precuneus subregions (7A vs. 7M) and Condition (Own Eyes vs. Observer)
[3] Interaction between precuneus subregions (7A vs. 7M) and Condition (Own Eyes vs. Retrieve)
[4] Interaction between precuneus subregions (7A vs. 7P) and Condition (Own Eyes vs. Observer)
[5] Interaction between precuneus subregions (7A vs. 7P) and Condition (Own Eyes vs. Retrieve)
[6] Interaction between Condition (Own Eyes vs. Observer) and percent signal change
[7] Interaction between Condition (Own Eyes vs. Retrieve) and percent signal change
[8] Interaction between precuneus subregions (7A vs. 7M) and percent signal change
[9] Interaction between precuneus subregions (7A vs. 7P) and percent signal change

Supplemental Table 16. Model Predicting Vividness from Precuneus (Probe Phase: Categorical Approach for Precuneus Subregions)

Fixed Effect	Estimate (β)	SE	95% CI Lower	95% CI Upper	t-value	p-value
Intercept	3.547	0.073	3.404	3.690	48.52	<.001
Retrieval Condition: Observer	-0.035	0.024	-0.082	0.011	-1.47	.141
Retrieval Condition: Retrieve	0.065	0.024	0.018	0.112	2.74	.006
Precuneus Subregion: 7M	-0.018	0.025	-0.067	0.030	-0.73	.463
Precuneus Subregion: 7P	-0.007	0.024	-0.055	0.041	-0.29	.769
% Sig. Change[1]	0.056	0.016	0.024	0.089	3.43	<.001
Precuneus Subregion x Condition[2]	-0.003	0.060	-0.121	0.114	-0.05	.956
Precuneus Subregion x Condition[3]	0.004	0.060	-0.113	0.122	0.07	.942
Precuneus Subregion x Condition[4]	-0.004	0.058	-0.119	0.110	-0.08	.935
Precuneus Subregion x Condition[5]	0.003	0.058	-0.111	0.118	0.06	.950
Condition x % Sig. Change[6]	0.009	0.034	-0.058	0.077	0.27	.784
Condition x % Sig. Change[7]	-0.013	0.034	-0.081	0.054	-0.40	.692
Precuneus Subregion x % Sig. Change[8]	-0.037	0.039	-0.115	0.040	-0.95	.343
Precuneus Subregion x % Sig. Change[9]	0.004	0.041	-0.075	0.084	0.11	.911
Vividness (S1 covariate)	0.231	0.029	0.173	0.290	7.77	<.001
Emotion (S1 covariate)	0.132	0.021	0.089	0.175	6.08	<.001
Perspective Bias (S1 covariate)	0.037	0.015	0.008	0.067	2.49	.020

Note. The reference level for the retrieval orientation factor is 'Own Eyes'. The reference level for the precuneus subregion factor is '7A'. Session 1 ratings were included as covariates to account for baseline variation in subjective memory experience.

[1] Percent signal change in the precuneus during the probe phase
[2] Interaction between precuneus subregions (7A vs. 7M) and Condition (Own Eyes vs. Observer)
[3] Interaction between precuneus subregions (7A vs. 7M) and Condition (Own Eyes vs. Retrieve)
[4] Interaction between precuneus subregions (7A vs. 7P) and Condition (Own Eyes vs. Observer)
[5] Interaction between precuneus subregions (7A vs. 7P) and Condition (Own Eyes vs. Retrieve)
[6] Interaction between Condition (Own Eyes vs. Observer) and percent signal change
[7] Interaction between Condition (Own Eyes vs. Retrieve) and percent signal change
[8] Interaction between precuneus subregions (7A vs. 7M) and percent signal change
[9] Interaction between precuneus subregions (7A vs. 7P) and percent signal change

Supplemental Table 17. Model Predicting Emotional Intensity from AG (Cue Phase: Categorical Approach for AG Subregions)

Fixed Effect	Estimate (β)	SE	95% CI Lower	95% CI Upper	t-value	p-value
Intercept	3.340	0.093	3.158	3.523	35.87	<.001
Retrieval Condition: Observer	-0.045	0.042	-0.128	0.037	-1.07	.287
Retrieval Condition: Retrieve	0.035	0.042	-0.047	0.118	0.84	.400
AG Subregion[1]	-0.000	0.024	-0.048	0.047	-0.03	.974
% Sig. Change[2]	-0.025	0.029	-0.083	0.031	-0.88	.380
AG Subregion x Condition[3]	0.002	0.060	-0.115	0.120	0.44	.965
AG Subregion x Condition[4]	0.003	0.059	-0.113	0.121	0.59	.953
Condition x % Sig. Change[5]	-0.031	0.054	-0.137	0.074	-0.58	.560
Condition x % Sig. Change[6]	-0.071	0.054	-0.177	0.034	-1.32	.188
AG Subregion x % Sig. Change	0.036	0.044	-0.049	0.123	0.83	.407
Vividness (S1 covariate)	0.061	0.027	0.007	0.114	2.23	.034
Emotion (S1 covariate)	0.426	0.033	0.361	0.491	12.88	<.001
Perspective Bias (S1 covariate)	-0.006	0.016	-0.037	0.025	-0.38	.707

Note. The reference level for the retrieval orientation factor is 'Own Eyes'. Session 1 ratings were included as covariates to account for baseline variation in subjective memory experience.

[1] PGa vs. PGp

[2] Percent signal change in left AG during the cue phase

[3] Interaction between left AG subregions and Condition (Own Eyes vs. Observer)

[4] Interaction between left AG subregions and Condition (Own Eyes vs. Retrieve)

[5] Interaction between Condition (Own Eyes vs. Observer) and percent signal change

[6] Interaction between Condition (Own Eyes vs. Retrieve) and percent signal change

Supplemental Table 18. Model Predicting Emotional Intensity from AG (Probe Phase: Categorical Approach for AG Subregions)

Fixed Effect	Estimate (β)	SE	95% CI Lower	95% CI Upper	t-value	p-value
Intercept	3.351	0.092	3.168	3.533	36.06	<.001
Retrieval Condition: Observer	-0.055	0.042	-0.138	0.028	-1.29	.197
Retrieval Condition: Retrieve	0.046	0.042	-0.037	0.130	1.08	.280
AG Subregion[1]	-0.027	0.025	-0.077	0.023	-1.06	.290
% Sig. Change[2]	0.103	0.048	0.008	0.198	2.13	.040
AG Subregion x Condition[3]	0.003	0.061	-0.117	0.123	0.05	.960
AG Subregion x Condition[4]	-0.007	0.060	-0.126	0.111	-0.12	.901
Condition x % Sig. Change[5]	-0.005	0.056	-0.116	0.105	-0.09	.926
Condition x % Sig. Change[6]	0.051	0.057	-0.059	0.163	0.90	.364
AG Subregion x % Sig. Change	-0.018	0.048	-0.113	0.077	-0.38	.707
Vividness (S1 covariate)	0.059	0.027	0.005	0.113	2.17	.038
Emotion (S1 covariate)	0.425	0.032	0.361	0.489	13.02	<.001
Perspective Bias (S1 covariate)	-0.004	0.016	-0.037	0.028	-0.26	.801

Note. The reference level for the retrieval orientation factor is 'Own Eyes'. Session 1 ratings were included as covariates to account for baseline variation in subjective memory experience.

[1] PGa vs. PGp

[2] Percent signal change in left AG during the probe phase

[3] Interaction between left AG subregions and Condition (Own Eyes vs. Observer)

[4] Interaction between left AG subregions and Condition (Own Eyes vs. Retrieve)

[5] Interaction between Condition (Own Eyes vs. Observer) and percent signal change

[6] Interaction between Condition (Own Eyes vs. Retrieve) and percent signal change

Supplemental Table 19. Model Predicting Emotional Intensity from Precuneus (Cue Phase: Categorical Approach for Precuneus Subregions)

Fixed Effect	Estimate (β)	SE	95% CI Lower	95% CI Upper	t-value	p-value
Intercept	3.345	0.092	3.163	3.526	36.12	<.001
Retrieval Condition: Own Eyes	-0.035	0.024	-0.083	0.012	-1.46	.144
Retrieval Condition: Observer	-0.079	0.024	-0.127	-0.032	-3.27	.001
Precuneus Subregion: 7M	0.004	0.024	-0.044	0.053	0.18	.855
Precuneus Subregion: 7P	-0.000	0.024	-0.048	0.047	-0.01	.995
% Sig. Change[1]	-0.012	0.021	-0.053	0.028	-0.59	.556
Precuneus Subregion x Condition[2]	-0.018	0.060	-0.136	0.099	-0.31	.760
Precuneus Subregion x Condition[3]	-0.022	0.060	-0.140	0.094	-0.38	.705
Precuneus Subregion x Condition[4]	0.003	0.059	-0.113	0.119	0.05	.958
Precuneus Subregion x Condition[5]	0.002	0.059	-0.113	0.119	0.05	.962
Condition x % Sig. Change[6]	0.090	0.036	0.019	0.161	2.51	.012
Condition x % Sig. Change[7]	0.091	0.036	0.021	0.162	2.54	.011
Precuneus Subregion x % Sig. Change[8]	0.006	0.040	-0.073	0.085	0.16	.874
Precuneus Subregion x % Sig. Change[9]	0.015	0.041	-0.065	0.097	0.38	.704
Vividness (S1 covariate)	0.063	0.026	0.012	0.115	2.42	.022
Emotion (S1 covariate)	0.422	0.032	0.358	0.486	12.95	<.001
Perspective Bias (S1 covariate)	-0.009	0.017	-0.044	0.025	-0.55	.586

Note. The reference level for the retrieval orientation factor is 'Retrieve'. The reference level for the precuneus subregion factor is '7A'. Session 1 ratings were included as covariates to account for baseline variation in subjective memory experience.

[1] Percent signal change in the precuneus during the cue phase
[2] Interaction between precuneus subregions (7A vs. 7M) and Condition (Retrieve vs. Own Eyes)
[3] Interaction between precuneus subregions (7A vs. 7M) and Condition (Retrieve vs. Observer)
[4] Interaction between precuneus subregions (7A vs. 7P) and Condition (Retrieve vs. Own Eyes)
[5] Interaction between precuneus subregions (7A vs. 7P) and Condition (Retrieve vs. Observer)
[6] Interaction between Condition (Retrieve vs. Own Eyes) and percent signal change
[7] Interaction between Condition (Retrieve vs. Observer) and percent signal change
[8] Interaction between precuneus subregions (7A vs. 7M) and percent signal change
[9] Interaction between precuneus subregions (7A vs. 7P) and percent signal change

Supplemental Table 20. Model Predicting Emotional Intensity from Precuneus (Probe Phase: Categorical Approach for Precuneus Subregions)

Fixed Effect	Estimate (β)	SE	95% CI Lower	95% CI Upper	t-value	p-value
Intercept	3.348	0.092	3.166	3.530	36.14	<.001
Retrieval Condition: Own Eyes	-0.034	0.024	-0.081	0.013	-1.40	.161
Retrieval Condition: Observer	-0.076	0.024	-0.123	-0.028	-3.11	.002
Precuneus Subregion: 7M	0.003	0.025	-0.047	0.053	0.13	.896
Precuneus Subregion: 7P	0.001	0.025	-0.047	0.051	0.08	.936
% Sig. Change[1]	-0.015	0.031	-0.077	0.046	-0.49	.629
Precuneus Subregion x Condition[2]	0.017	0.060	-0.101	0.136	0.29	.772
Precuneus Subregion x Condition[3]	0.044	0.060	-0.074	0.164	0.73	.463
Precuneus Subregion x Condition[4]	0.002	0.059	-0.113	0.118	0.05	.964
Precuneus Subregion x Condition[5]	0.013	0.059	-0.102	0.130	0.23	.819
Condition x % Sig. Change[6]	-0.049	0.035	-0.119	0.019	-1.41	.158
Condition x % Sig. Change[7]	-0.107	0.035	-0.177	-0.037	-3.02	.003
Precuneus Subregion x % Sig. Change[8]	-0.001	0.040	-0.081	0.078	-0.04	.966
Precuneus Subregion x % Sig. Change[9]	-0.004	0.041	-0.086	0.077	-0.11	.911
Vividness (S1 covariate)	0.063	0.026	0.012	0.115	2.43	.022
Emotion (S1 covariate)	0.422	0.032	0.358	0.486	12.86	<.001
Perspective Bias (S1 covariate)	-0.007	0.016	-0.040	0.025	-0.46	.648

Note. The reference level for the retrieval orientation factor is 'Retrieve'. The reference level for the precuneus subregion factor is '7A'. Session 1 ratings were included as covariates to account for baseline variation in subjective memory experience.

[1] Percent signal change in the precuneus during the probe phase
[2] Interaction between precuneus subregions (7A vs. 7M) and Condition (Retrieve vs. Own Eyes)
[3] Interaction between precuneus subregions (7A vs. 7M) and Condition (Retrieve vs. Observer)
[4] Interaction between precuneus subregions (7A vs. 7P) and Condition (Retrieve vs. Own Eyes)
[5] Interaction between precuneus subregions (7A vs. 7P) and Condition (Retrieve vs. Observer)
[6] Interaction between Condition (Retrieve vs. Own Eyes) and percent signal change
[7] Interaction between Condition (Retrieve vs. Observer) and percent signal change
[8] Interaction between precuneus subregions (7A vs. 7M) and percent signal change
[9] Interaction between precuneus subregions (7A vs. 7P) and percent signal change

Supplemental Table 21. Model Predicting Perspective Maintenance from AG (Cue Phase: Categorical Approach for AG Subregions)

Fixed Effect	Estimate (β)	SE	95% CI Lower	95% CI Upper	t-value	p-value
Intercept	3.683	0.090	3.507	3.860	40.87	<.001
Retrieval Condition: Observer	-0.332	0.052	-0.436	-0.229	-6.29	<.001
Retrieval Condition: Retrieve	0.116	0.052	0.012	0.219	2.21	.027
AG Subregion[1]	-0.003	0.030	-0.063	0.055	-0.13	.900
% Sig. Change[2]	0.045	0.036	-0.026	0.116	1.24	.215
AG Subregion x Condition[3]	0.015	0.074	-0.131	0.162	0.21	.834
AG Subregion x Condition[4]	0.012	0.074	-0.133	0.158	0.17	.864
Condition x % Sig. Change[5]	-0.141	0.067	-0.273	-0.008	-2.09	.037
Condition x % Sig. Change[6]	-0.162	0.067	-0.294	-0.030	-2.41	.016
AG Subregion x % Sig. Change	-0.051	0.054	-0.159	0.056	-0.94	.349
Vividness (S1 covariate)	0.064	0.026	0.011	0.117	2.39	.024
Emotion (S1 covariate)	0.056	0.024	0.009	0.104	2.35	.027
Perspective Bias (S1 covariate)	0.033	0.018	-0.001	0.069	1.86	.077

Note. The reference level for the retrieval orientation factor is ‘Own Eyes’. Session 1 ratings were included as covariates to account for baseline variation in subjective memory experience.

[1] PGa vs. PGp

[2] Percent signal change in left AG during the cue phase

[3] Interaction between left AG subregions and Condition (Own Eyes vs. Observer)

[4] Interaction between left AG subregions and Condition (Own Eyes vs. Retrieve)

[5] Interaction between Condition (Own Eyes vs. Observer) and percent signal change

[6] Interaction between Condition (Own Eyes vs. Retrieve) and percent signal change

Supplemental Table 22. Model Predicting Perspective Maintenance from AG (Probe Phase: Categorical Approach for AG Subregions)

Fixed Effect	Estimate (β)	SE	95% CI Lower	95% CI Upper	t-value	p-value
Intercept	3.689	0.088	3.514	3.863	41.50	<.001
Retrieval Condition: Observer	-0.329	0.053	-0.434	-0.224	-6.16	<.001
Retrieval Condition: Retrieve	0.111	0.053	0.006	0.216	2.07	.038
AG Subregion[1]	0.000	0.031	-0.061	0.062	0.02	.983
% Sig. Change[2]	-0.004	0.052	-0.107	0.099	-0.08	.940
AG Subregion x Condition[3]	0.002	0.077	-0.148	0.153	0.03	.974
AG Subregion x Condition[4]	0.022	0.076	-0.126	0.171	0.30	.764
Condition x % Sig. Change[5]	-0.026	0.070	-0.164	0.111	-0.38	.704
Condition x % Sig. Change[6]	-0.077	0.070	-0.215	0.061	-1.09	.277
AG Subregion x % Sig. Change	0.083	0.059	-0.033	0.200	1.39	.164
Vividness (S1 covariate)	0.066	0.026	0.014	0.118	2.49	.020
Emotion (S1 covariate)	0.054	0.024	0.008	0.102	2.29	.030
Perspective Bias (S1 covariate)	0.028	0.018	-0.007	0.064	1.57	.131

Note. The reference level for the retrieval orientation factor is 'Own Eyes'. Session 1 ratings were included as covariates to account for baseline variation in subjective memory experience.

[1] PGa vs. PGp

[2] Percent signal change in left AG during the probe phase

[3] Interaction between left AG subregions and Condition (Own Eyes vs. Observer)

[4] Interaction between left AG subregions and Condition (Own Eyes vs. Retrieve)

[5] Interaction between Condition (Own Eyes vs. Observer) and percent signal change

[6] Interaction between Condition (Own Eyes vs. Retrieve) and percent signal change

Supplemental Table 23. Model Predicting Perspective Maintenance from Precuneus (Cue Phase: Categorical Approach for Precuneus Subregions)

Fixed Effect	Estimate (β)	SE	95% CI Lower	95% CI Upper	t-value	p-value
Intercept	3.686	0.090	3.509	3.864	40.76	<.001
Retrieval Condition: Own Eyes	-0.322	0.030	-0.381	-0.262	-10.57	<.001
Retrieval Condition: Observer	0.126	0.030	0.067	0.186	4.18	<.001
Precuneus Subregion: 7M	-0.013	0.031	-0.074	0.046	-0.45	.654
Precuneus Subregion: 7P	-0.004	0.030	-0.063	0.055	-0.13	.893
% Sig. Change[1]	0.044	0.030	-0.015	0.103	1.45	.158
Precuneus Subregion x Condition[2]	0.023	0.075	-0.123	0.170	0.31	.755
Precuneus Subregion x Condition[3]	0.015	0.074	-0.130	0.162	0.21	.832
Precuneus Subregion x Condition[4]	-0.006	0.074	-0.151	0.139	-0.08	.935
Precuneus Subregion x Condition[5]	-0.001	0.073	-0.146	0.142	-0.03	.979
Condition x % Sig. Change[6]	-0.120	0.045	-0.208	-0.032	-2.67	.008
Condition x % Sig. Change[7]	-0.066	0.045	-0.154	0.021	-1.48	.140
Precuneus Subregion x % Sig. Change[8]	-0.076	0.050	-0.175	0.023	-1.51	.132
Precuneus Subregion x % Sig. Change[9]	-0.066	0.051	-0.168	0.035	-1.28	.200
Vividness (S1 covariate)	0.058	0.027	0.005	0.111	2.16	.040
Emotion (S1 covariate)	0.057	0.025	0.007	0.106	2.27	.032
Perspective Bias (S1 covariate)	0.029	0.018	-0.006	0.065	1.59	.127

Note. The reference level for the retrieval orientation factor is ‘Own Eyes’. The reference level for the precuneus subregion factor is ‘7A’. Session 1 ratings were included as covariates to account for baseline variation in subjective memory experience.

[1] Percent signal change in the precuneus during the cue phase

[2] Interaction between precuneus subregions (7A vs. 7M) and Condition (Own Eyes vs. Observer)

[3] Interaction between precuneus subregions (7A vs. 7M) and Condition (Own Eyes vs. Retrieve)

[4] Interaction between precuneus subregions (7A vs. 7P) and Condition (Own Eyes vs. Observer)

[5] Interaction between precuneus subregions (7A vs. 7P) and Condition (Own Eyes vs. Retrieve)

[6] Interaction between Condition (Own Eyes vs. Observer) and percent signal change

[7] Interaction between Condition (Own Eyes vs. Retrieve) and percent signal change

[8] Interaction between precuneus subregions (7A vs. 7M) and percent signal change

[9] Interaction between precuneus subregions (7A vs. 7P) and percent signal change

Supplemental Table 24. Model Predicting Perspective Maintenance from Precuneus (Probe Phase: Categorical Approach for Precuneus Subregions)

Fixed Effect	Estimate (β)	SE	95% CI Lower	95% CI Upper	t-value	p-value
Intercept	3.697	0.091	3.518	3.877	40.41	<.001
Retrieval Condition: Own Eyes	-0.365	0.054	-0.472	-0.257	-6.66	<.001
Retrieval Condition: Observer	0.069	0.055	0.038	0.178	1.26	.207
Precuneus Subregion: 7M	-0.006	0.032	-0.069	0.056	-0.21	.834
Precuneus Subregion: 7P	-0.011	0.031	-0.072	0.049	-0.37	.708
% Sig. Change[1]	0.054	0.050	-0.043	0.152	1.10	.275
Precuneus Subregion x Condition[2]	0.035	0.077	-0.116	0.186	0.45	.651
Precuneus Subregion x Condition[3]	0.035	0.075	-0.112	0.184	0.47	.635
Precuneus Subregion x Condition[4]	0.064	0.077	-0.086	0.216	0.84	.401
Precuneus Subregion x Condition[5]	0.042	0.076	-0.107	0.192	0.56	.577
Condition x % Sig. Change[6]	-0.239	0.101	-0.438	-0.040	-2.36	.018
Condition x % Sig. Change[7]	-0.312	0.102	-0.513	-0.110	-3.04	.002
Precuneus Subregion x % Sig. Change[8]	-0.091	0.050	-0.191	0.007	-1.80	.071
Precuneus Subregion x % Sig. Change[9]	-0.014	0.052	-0.116	0.087	-0.28	.779
Precuneus Subregion x % Sig. Change x Condition[10]	0.280	0.119	0.047	0.514	2.35	.019
Precuneus Subregion x % Sig. Change x Condition[11]	0.154	0.125	-0.090	0.399	1.24	.217
Precuneus Subregion x % Sig. Change x Condition[12]	0.266	0.119	0.032	0.501	2.23	.026
Precuneus Subregion x % Sig. Change x Condition[13]	0.162	0.126	-0.084	0.409	1.29	.197
Vividness (S1 covariate)	0.059	0.027	0.006	0.112	2.19	.038
Emotion (S1 covariate)	0.058	0.024	0.010	0.106	2.39	.024
Perspective Bias (S1 covariate)	0.028	0.019	-0.008	0.066	1.52	.144

Note. The reference level for the retrieval orientation factor is 'Own Eyes'. The reference level for the precuneus subregion factor is '7A'. Session 1 ratings were included as covariates to account for baseline variation in subjective memory experience.

[1] Percent signal change in the precuneus during the probe phase

[2] Interaction between precuneus subregions (7A vs. 7M) and Condition (Own Eyes vs. Observer)

[3] Interaction between precuneus subregions (7A vs. 7M) and Condition (Own Eyes vs. Retrieve)

[4] Interaction between precuneus subregions (7A vs. 7P) and Condition (Own Eyes vs. Observer)

[5] Interaction between precuneus subregions (7A vs. 7P) and Condition (Own Eyes vs. Retrieve)

[6] Interaction between Condition (Own Eyes vs. Observer) and percent signal change
[7] Interaction between Condition (Own Eyes vs. Retrieve) and percent signal change
[8] Interaction between precuneus subregions (7A vs. 7M) and percent signal change
[9] Interaction between precuneus subregions (7A vs. 7P) and percent signal change
[10] Three-way interactions between precuneus subregions (7A vs.7M), percent signal change, and condition (Own Eyes vs. Observer)
[11] Three-way interactions between precuneus subregions (7A vs.7P), percent signal change, and condition (Own Eyes vs. Observer)
[12] Three-way interactions between precuneus subregions (7A vs.7M), percent signal change, and condition (Own Eyes vs. Retrieve)
[13] Three-way interactions between precuneus subregions (7A vs.7P), percent signal change, and condition (Own Eyes vs. Retrieve)

Supplemental Table 25

Descriptive statistics in Session 1 and Session 2

	Own Eyes	Observer	Retrieve
**Session 1**			
Memory Age (years)	9.92 (1.77)	9.89 (1.89)	9.88 (1.92)
Vividness	3.65 (.56)	3.65 (.55)	3.61 (.54)
Emotional Intensity	3.33 (.54)	3.28 (.58)	3.28 (.56)
Positive Valence	3.41 (.38)	3.35 (.36)	3.39 (.44)
Own Eyes Perspective	3.77 (.73)	3.76 (.73)	3.80 (.72)
Observer Perspective	2.18 (.70)	2.18 (.71)	2.15 (.70)
**Session 2**			
Vividness	3.53 (.61)	3.49 (.59)	3.59 (.55)
Vividness RT (s)	1.26 (.25)	1.26 (.26)	1.21 (.25)
Emotional Intensity	3.31 (.66)	3.25 (.70)	3.33 (.63)
Emotional Intensity RT (s)	1.33 (.31)	1.34 (.27)	1.31 (.22)
Perspective Maintenance	3.74 (.69)	3.42 (.61)	3.86 (.60)
Perspective Maintenance RT (s)	1.18 (.33)	1.23 (.33)	1.24 (.34)

Note. *Mean* (*SD*)

Supplemental Table 26

Regions Commonly Activated in the Conjunction Analysis for the Cue and Probe Phases (Own Eyes > Retrieve ∩ Observer > Retrieve)

				MNI peak		
Region	Voxels	BA	t	x	y	z
**Cue**						
Visual Cortex	170	19	4.54	-42	-76	22
		19	3.32	-38	-66	18
Angular Gyrus		39	4.08	-48	-70	18
**Probe**						
no suprathreshold clusters						

MNI = Montreal Neurological Institute; BA = Brodmann's Area;

p = .001, FWEc = 170.

Supplemental Table 27

Results of the Paired Samples T-test for the Cue and Probe Phases

				MNI peak		
Region	Voxels	BA	t	x	y	z
**Cue**						
**Own Eyes > Observer & Retrieve**						
no suprathreshold clusters						
**Observer > Own Eyes & Retrieve**						
Visual Cortex	487	18	6.52	26	-92	2
		18	6.01	26	-98	-6
		18	5.13	32	-94	8
	366	18	5.57	-18	-104	-6
		18	5.46	-26	-100	4
		18	5.25	-26	-100	-8
**Probe**						
**Own Eyes > Observer & Retrieve**						
no suprathreshold clusters						
**Observer > Own Eyes & Retrieve**						
Posterior Cingulate Cortex	265	31	6.58	-8	-60	44
		31	4.33	0	-54	40
Precuneus		7	3.59	0	-70	50
Anterior Prefrontal Cortex	281	10	5.54	36	52	-4

		10	4.70	24	48	2
		10	4.30	26	62	-6
	163	10	5.12	-32	52	8
		10	4.90	-30	52	-4
		10	4.30	-30	60	-2
Angular Gyrus	116	39	5.03	-40	-80	38
Visual Cortex		19	4.77	-40	-84	30
		19	4.35	-40	-74	26

MNI = Montreal Neurological Institute; BA = Brodmann's Area;

Cue: p = .001, FWEc = 366. Probe: p = .001, FWEc = 116.

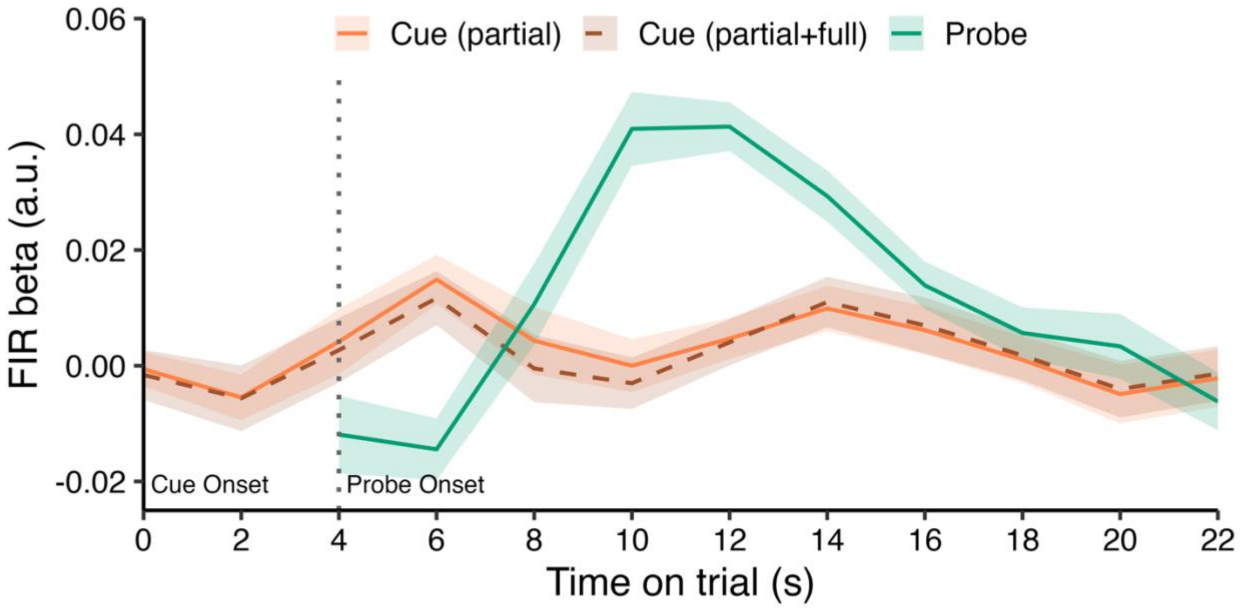

Supplemental Figure 1. Finite impulse response (FIR) timecourses (2-s bins, mean ± 95% CI) averaged across conditions (Own-Eyes, Observer, Retrieve) in the left angular gyrus to illustrate temporal separation of cue- and probe-related activity. Cue responses (solid orange line) were estimated from partial trials, showing a distinct early peak at ~6 s. Probe responses (solid teal line, shifted to trial time for display) exhibited a later and larger peak at ~10–12 s. The dashed brown line shows cue-related activity when combining partial and full trials, which closely overlaps with cue-only estimates in the early bins. This demonstrates that including both partial and full trials in the model allows reliable separation of cue- and probe-related responses despite their temporal proximity. The vertical dotted line marks the probe onset (4 s).

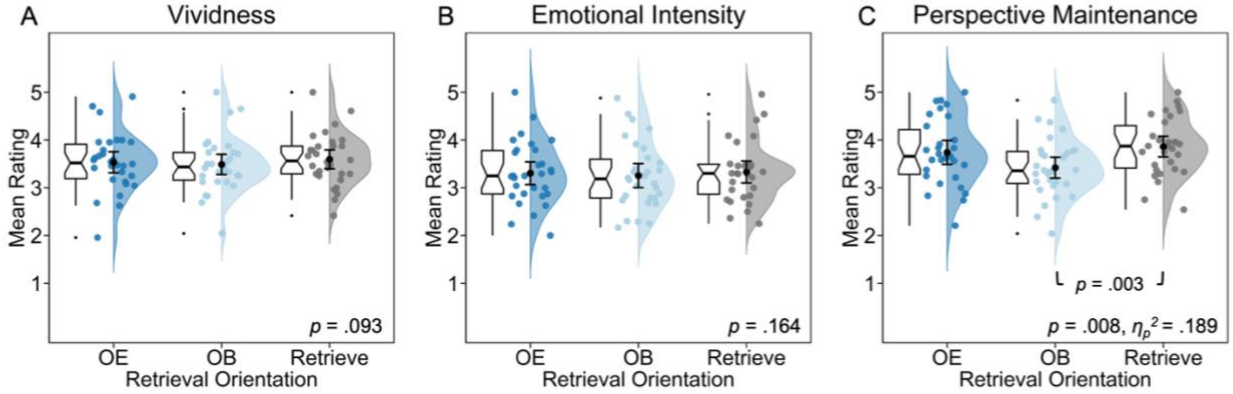

Supplemental Figure 2. Online behavioral ratings during fMRI scanning (Session 2), averaged within each participant. Results showed no significant difference for (A) Vividness and (B) Emotional Intensity. However, participants were better able to (C) maintain their natural perspective in the Retrieve condition, compared to remembering AMs from an observer-like perspective.

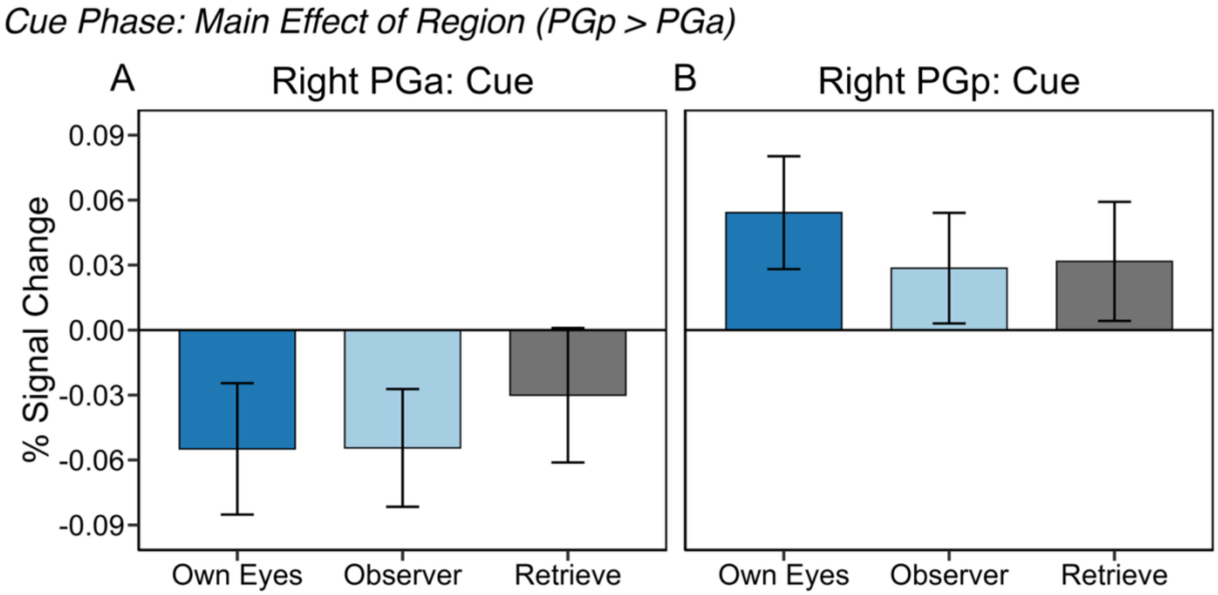

Supplemental Figure 3. ROI analysis in right angular gyrus (AG) for PGa (A) and PGp (B).
